# Names and “Doing Gender”: How Forenames and Surnames Contribute to Gender Identities, Difference, and Inequalities

**DOI:** 10.1007/s11199-017-0805-4

**Published:** 2017-07-10

**Authors:** Jane Pilcher

**Affiliations:** School of Media, Communication and Sociology, University of Leicester, Bankfield House, 132 New Walk, Leicester, LE1 7JA UK

**Keywords:** Naming practices, Doing gender, Gendered embodied named identity, Forenames, Surnames, Identities, Inequalities

## Abstract

Names, as proper nouns, are clearly important for the identification of individuals in everyday life. In the present article, I argue that forenames and surnames need also to be recognized as “doing” words, important in the categorization of sex at birth and in the ongoing management of gender conduct appropriate to sex category. Using evidence on personal naming practices in the United States and United Kingdom, I examine what happens at crisis points of sexed and gendered naming in the life course (for example, at the birth of babies, at marriage, and during gender-identity transitions). I show how forenames and surnames help in the embodied doing of gender and, likewise, that bodies are key to gendered practices of forenaming and surnaming: we have “gendered embodied named identities.” Whether normative and compliant, pragmatic, or creative and resistant, forenaming and surnaming practices are revealed as core to the production and reproduction of binary sex categories and to gendered identities, difference, hierarchies, and inequalities.

Governed as they are by common law and national or local statutes, practices of personal naming in the United Kingdom and United States are relatively unfettered by legislation (for the U.S., see MacDougall 1985; for the UK, see Finch 2008). Nonetheless, practices of personal naming in these countries are patterned and structured: first, as a consequence of replication of usage of the names of individuals and recurring demands for the authentication of individual identity over time; second, by cultural traditions and conventions (including those patrilineal and patriarchal in origin) whereby personal names are used to mark individual and social identities (Finch 2008). Despite their fundamental and ubiquitous importance for each individual in a multitude of contexts, scholars are only just beginning to give personal names and naming practices “the theoretical and analytical scrutiny” they deserve (Palsson 2014, p. 618).

In particular, sex and gendered forenaming and surnaming practices suffer from a form of what psychologists call *functional fixedness*, whereby the well-known function of an object masks its other possible uses (Corsini 1999). In the case of personal names, their everyday familiarity makes it hard to move beyond a taken-for-granted understanding of their use and meaning within the gendered social world. My aim in the present article is to break down this functional fixedness about personal names and gender and thereby achieve a step-change in the theoretical and analytical scrutiny of their relationship.

The sociologist Norbert Elias (1991) argued that forenames help denote individuality (or “I” identities), whereas surnames are important in signaling “We” identities (i.e., “We are family”). For sure, forenames-plus-surnames operate in these kinds of ways simultaneously to display both individuality and connectedness. In so doing, though, it is my argument in the present article that forenames and surnames are also constructing and displaying sex and gender. The display of individuality and connectedness through personal names is a *gendered* process and is especially apparent when newborns are given forenames, when transgender people choose forenames for themselves, when heterosexual women marry, and when children are given surnames. In an innovative approach, I critically re-evaluate evidence in relation to these personal naming practices and anchor it in an original theoretical configuration in which I deploy my own concept of “gendered embodied named identity” (see also Pilcher 2016, p. 774), as well as draw on the “doing gender” perspective (Kessler and McKenna 1978; West and Zimmerman 1987) and the framework of “habits, crises and creativity” offered by Shilling (2008, p. 1). Using this approach, in the final section of my article I identify the multifaceted uses of personal names and their socially consequentiality in the context of the power structures of sex, gender, and race/ethnicity in societies such as the United Kingdom and United States.

## Names and (Re)Doing Gender

Sociological understandings of gender as doing derive both from Goffman’s symbolic interactionist analysis of gender display (for example, Goffman 1959) and Garfinkel’s (1967) ethnomethodology of gender, with subsequent elaborations by Kessler and McKenna (1978) and West and Zimmerman (1987). In summary, in the doing gender approach, both sex and gender are theorized as socially constructed categorizations, identifications, and practices—routinely accomplished in everyday, ongoing social interactions and within which bodies are central. More recently, this approach has piqued sociological interest in the “re-doing” of gender, where the embodied identifications and practices of individuals deviate from normative sex categorizations and gender displays (for example, see Connell 2010). Together, these features make the doing gender approach useful for my purposes in the present article. Also useful for my purposes is the work of Shilling (2008). In his discussion of processes and changes that arise from the “habits, crises and creativity” (p. 1) swirling in and out of people’s lives over time and affecting their bodies and identities, Shilling bestows a framework I can use to explore naming practices at critical points across the life course of individuals and families, including at birth and at marriage.

My summary of Shilling’s work and the doing gender perspective shows that embodiment and identities are recognized by scholars to be inextricably intertwined (Howson 2004; Jenkins 2008; Lawler 2008; Shilling 2008, 2012). However, as I have argued elsewhere (Pilcher 2016), our identities inextricably involve not only our bodies but also our names. Consequently, the concept of *embodied named identity* is necessary to capture the outcomes of identification practices of naming that are fundamentally orientated around and rooted in the body. In the present article, my focus is more specifically on the nexus of names, sexed and gendered bodies, and identities. In other words, my focus is on gendered embodied named identities. I show how sexed and gendered bodies are irreducibly intertwined with the identity practices of personal naming: Bodies are key to gendered practices of forenaming and surnaming, and forenames and surnames likewise help in the embodied doing of gender.

In the remainder of my article, I re-evaluate a range of evidence on sex, gender, and naming practices in relation to three key questions. First, how do embodied individuals respond to routinized naming traditions in relation to sex and gender at critical/crisis points of naming in the life course (for example, newborn babies, marriage, divorce, gender-identity transitions)? Second, how do individuals experience and manage crises brought about by the breaching of normative expectations about the coincidence of names with sexed/gendered bodies? Third, in what ways are naming practices and their outcomes socially uneven and unequal (Duncan 2011)?

## Children’s Forenaming

Contemporary reproductive technologies allow the sexing of a fetus (categorizing its sex) to occur prior to birth. Whether before or after its birth, a child’s sex is decided through the application of socially agreed biological criteria (typically using the appearance of genitalia) for classifying bodies as female or male. Once the child has been sexed using the body, parents must, shortly thereafter, make a choice about the forename their child will have. Determinants of forename choices made for children include kin and ethnic affiliations and honoring (discussed further in the following), as well as changing cultural influences (Alford 1988; Daly and Wilson 1982; Edwards and Caballero 2008; Fryer and Levitt 2003; Lieberson 2000; Sue and Telles 2007). Yet, it is the sex categorization of the child as female or male which remains the primary determinant of forename choices.

It is a very strong cultural norm in the United Kingdom and United States (as elsewhere) to give a new-born baby a sex-specific forename (Alford 1988), according to the sex categorization of its body as male or female. Androgynous, or sex/gender-neutral forenames, are rarely used. In an analysis of U.S. data by Lieberson et al. (2000), 97% of forenames regarded as female-appropriate are only given to children whose sex category is female. Likewise, 97% of forenames viewed as male-appropriate are only given to children whose sex category is male (see also Herbert and Aylene 2014). As Lieberson and colleagues’ analysis shows, there was no significant increase in the use of androgynous forenames in the twentieth century despite the important changes in gender relations that occurred during that period. Indeed, books and websites of today advising parents-to-be on baby names still tend to list possibilities only by the sex categories of boys and girls (for example, www.babynames.com; Joynes 2013). Moreover, in its annual report on the most popular baby forenames, the UK’s Office of National Statistics (2015) list forenames only by two sex categories (boys and girls).

At the very beginning of the incorporation of a newborn into social personhood, then, forenames are used in the decisive accomplishment of the categorization of sex (e.g., This baby has a vagina, therefore it is female. So, she will have a girl’s name.) and in the management of gender conduct appropriate to sex category (e.g., You can know this baby is female even when her genitals are hidden because the forename given to her clearly displays her sex category). As argued by Messerschmidt (2009), sex-categorized bodies are necessarily involved in the doing of gender, including in relation to my own example of forenaming practices. Once the initial sex-specific forenaming has been made, the given boy’s or girl’s forename acts (in most cases) repeatedly to categorize the individual as being a female or male throughout their subsequent life course.

As my illustrative examples of forenaming show, gendered embodied named identity is a core part of what Shilling (2008, p. 15) calls the “body pedagogy” of an individual’s appearance, activities, skills, and capacities which bolster claims to their membership in a sex category and cumulatively and constantly accomplish their gender. Because there are strong normative expectations about the coincidence of body pedagogy and forenames (Alford 1988; Lieberson et al. 2000), any breaches may affect claims to membership in a sex category and disrupt the performance and accomplishment of gender. Useful here is Connell’s (2009, p. 107) concept of “contradictory embodiment,” which describes aspects of embodiment that are perceived to be abnormal or irregular (also see Messerschmidt 2009).

In relation to personal names, I argue that *contradictory embodiment* occurs when normative expectations about the coincidence of bodies, sex category, gender, and forenames are breached. If individuals are perceived to have (whether by themselves or by others) a gender-wrong forename, a person’s femininity or masculinity may be disrupted as a consequence. The song “A Boy Named Sue” (popularized by country singer Johnny Cash) details the gender trouble that stems from the contradictory embodiment of a boy having a female-appropriate forename (Absolute Lyrics 2017). This fictional portrayal of the consequences of contradictory embodiment in relation to forenames is supported by Figlio’s (2007) research. In this U.S.-based study of behavior problems and students’ test scores, it was found that, in middle school, a large gap emerged in behavior between boys with female-typed names and other boys; specifically, the former were more disruptive in school than the latter were.

To explain why boys with gender-inappropriate forenames may experience problems and their gender practices held to account, I can apply Thorne’s (1993) concept of *gender contamination*. In a classic study of gender play, Thorne claims that girls who are tomboys gain benefit from associating with masculinities, but boys who behave or look like girls (or, as in my example, have a “girl’s” forename) are contaminated by their association with lesser valued femininities. Similarly, in their account of patterns of change in androgynous forenames (whereby increased deployment for girls leads to decreased deployment for boys), Lieberson et al. (2000, p. 1285) argue that the “advantaged group [boys] have more to lose in symbolic terms when their distinctive features are merged with the less advantaged population,” that is, girls.

Correspondingly, there is also some evidence that girls with typically boys’ forenames may gain some advantage from their contradictory embodiment. Their (name-based) association with higher-valued masculinities may mean, for example, an increased likelihood of studying science and mathematics (Bryner 2010; see also Coffey and McLaughlin 2009). These findings suggest that a girl with a male-appropriate forename may benefit from that contradictory embodiment, whereas a boy with a female-typed forename is likely to be contaminated by it. Moreover, Moss-Racusin et al.’s (2012) study suggests that, for girls and women, having a forename that *is* recognizably typical for their sex category and gender can be disadvantageous in some circumstances. In a study of gender bias among science faculty staff seeking to hire a laboratory manager, Moss-Racusin and colleagues showed that, in otherwise identical job applications, candidates with the sex-typed forename of John were rated more highly than were candidates with the sex-typed forename of Jennifer. So, in addition to marking and displaying embodied sex category, I argue that forenames are important in the doing of gender in terms of maintaining a gender hierarchy in which masculinities are routinely ranked over and above femininities.

Forenames are also important, I argue, in gendered embodied practices of “doing difference” (West and Fenstermaker 1995) with regard to race and ethnicity. Studies have shown that forenaming practices are important in relation to what I see as embodied processes of racial and ethnic honoring and identification. For example, in a UK-based study, Edwards and Caballero (2008) found that mixed-heritage parent couples wanted forenames that symbolized their children’s heritage in relation to race, ethnicity, and/or faith, alongside signaling their children’s (in my terms) embodied sex category and gender. Using data that covered every child born in the U.S. state of California since the 1960s and 1970s, Fryer and Levitt’s (2003) analysis of forenames by gender and race documents some marked differences between choices of Blacks and Whites. Some forenames (such as Shanice and Precious) were found to be relatively popular among Blacks for girls, but virtually unheard of for White girls; some boys’ forenames (such as Connor and Jake) were found to be distinctively White, with less than 2% of those so forenamed being Black.

Another U.S.-based study suggests that sex and gender matter in terms of the ways embodied forename choices can signal difference from and assimilation to majority cultures. Sue and Telles (2007) examined the forenaming practices of Latino/a parents in relation to gender. Hispanic couples tended to give their male children Spanish-appropriate forenames, but more often gave their female children English-typed forenames. This patterning of forenames, sex, gender, and Hispanic ethnicity was argued to be a reflection of boys being regarded as the key carriers of ethnic tradition and as less in need of protection against racial discrimination than were girls.

Similarly in the United Kingdom, the Office for National Statistics’ annual report (2015) on the most popular baby names is also suggestive of the ways that forenames for boys may signal ethnic credentials or heritage more often than forenames for girls do. The latest data show that Muhammad was the 12th most popular name given to baby boys in England and Wales in 2015. Within Muslim families, the forename Muhammad (or one of its spelling variations) is very dominant because it is traditional to name baby boys after the prophet of Islam in order to honor and respect him. As noted by Arnett (2014), there is a lower variance in boys’ forenames among UK’s Muslims than among other communities. In contrast, data (Office for National Statistics 2015) do not indicate equivalent practices among Muslim families in England and Wales when forenaming girls.

Such evidence on race, ethnicity, and forenaming practices is suggestive of the ways cultural processes of embodied identification relating both to the persistence of collective identity and/or its transcendence (doing difference) through the forenames chosen for children are at the same time heavily reliant on doing gender. Analysis of forenaming practices in relation to race and ethnicity are also revealing of inequalities in a gender order whereby boys may have a more enhanced status as embodied carriers of ethnic heritage and bearers of ethnic credentials compared to girls. Clearly, forenames can do important cultural work in relation to doing difference. The concept of gendered embodied named identity helps us to recognize that the important cultural work forenames do around difference is (strongly and at the same time) also about doing sex and gender.

## Transgender People’s Forenaming

The laissez-faire character of the UK and U.S. legal frameworks regulating personal names means, of course, that an individual unhappy with their “gender wrong” or “ethnic wrong” forename can change it during their life course to something they feel to be more right for themselves. For example, the UK writer and broadcaster Clive James opted to change from his given forename, Vivien, which is widely regarded in English-speaking countries as a female-appropriate name (McCrum 2013), whereas the U.S. sportsman Cassius Clay opted to change his name to Muhammad Ali in 1964 following his conversion to Islam (Peter 2016).

For transgender people, forenames are very obviously intertwined with the embodiment of sex and gender and, like these individuals’ visual appearance, are an important part of their purposeful “redoing” of their sex category and gender display. Well-known examples of transgender people changing their forenames in relation to their redoing of gender include Chelsea Elizabeth Manning, formerly Bradley Manning, a U.S. soldier jailed in 2013 for leaking classified documents (Gabbatt 2013). In changing their gender-wrong birth forename to a sex-specific and/or gender-right one that is appropriate to their chosen gender, forenames are an important element of the efforts of some transgender people to pass in everyday interactions.

Forenames also act as a fundamental component of evidence of gender change in documents and other important cultural artifacts for identification that support and confirm the new, civil-legal identity of transgender people. For those transgender people who identify as gender queer, a conscious choice of a forename that is sex- and gender-neutral helps present and authenticate their gender identity as one that does not adhere to the binaries of female/male or feminine/masculine. In Connell’s (2010) study, the interviewee Agape is argued to have chosen a gender-neutral forename precisely because it underscored Agape’s resistance to the gender binary and was an important tool in Agape’s conscious redoing of gender.

In addition to Agape, Connell (2010) recounts the experiences of another of her interviewees, Julie, a male-to-female transgender person who worked in telephone-based customer service. Julie found that customers often tried to make sense of her masculine-sounding voice by “mishearing” her forename and changing it to a “guy’s name” (p.41). Julie’s experiences are revealing, I argue, in at least two respects. First, they suggest the strength of cultural expectations that forenames correctly signal and match the embodiment of sex category (in this case, a masculine-sounding voice) and, second, they are illustrative of contradictory embodiment and of the strategies individuals use (in this case, mishearing a forename and substituting it with a sex- and gender-appropriate one) so as to repair and sustain their common-sense knowledge about the congruity of sex, gender, and forenames.

Julie’s telephone interactions with customers illuminate the part played by forenames in the doing of gender: Forenames are key to the social practices of placing others in gender categories (“determining gender” as Westbrook and Schilt 2014, p. 32, term it) and to processes whereby individuals are held to be accountable to sex category membership (West and Zimmerman 2009). Forenames, then, have a key role in presenting and authenticating individuals as belonging to the assumed binary sex category of either female or male and are important in the interpretation of the individual’s identity as authentically either feminine or masculine. Consequently, transgender people and gender queers are particularly likely to have to deal with the consequences if their forename is perceived by others to contradict their embodiment, that is, to “inaccurately” or “unclearly” declare and display their sex category and their gender.

The examples of the forenaming of children (including in relation to doing ethnic difference) and forenaming by transgender people have allowed me to show that sexed and gendered forenaming deserves greater attention by gender scholars and that the concept of gendered embodied named identity has great utility in this respect. In relation to embodied sex and gender, forenaming is a primary tool of sex categorization and of gender display—acting to reinforce “particular images of femininity and masculinity” (Shilling 2012, p.108), including in socially uneven and unequal ways. Next, I examine the ways surnaming practices are related to sex categorization and contribute to the (re)doing of gender.

## Marital and Children’s Surnaming

As my critical evaluation of the literature has shown, relatively few sociological studies have focused primarily on forenames and gender. In contrast, studies of heterosexual women’s surname choices at marriage are more plentiful (e.g., Boxer and Gritsenko 2005; Goldin and Shim 2004; Gooding and Kreider 2010; Hamilton et al. 2011; Lockwood et al. 2011; Mills 2003; Robnett et al. 2016; Scheuble et al. 2012; Shafer 2017; Twenge 1997; Valetas 2001; Wilson 2009). There are also several studies of surname choices made for children (Almack 2005; Davies 2011; Dempsey and Lindsay 2017; Johnson and Scheuble 2002; Lockwood et al. 2011; Nugent 2010). This body of work primarily documents patterns of change and determinants of choice or is undertaken from the perspective of family and kinship practices. As such, it is less concerned with how naming contributes to social practices of sex and gender.

In this section of my article, I repurpose the body of work on heterosexual women’s surname choices and on children’s surnames to explore the roles surnames play in the doing and redoing of gender. (As I explain in the following, there is a paucity of evidence on surnaming practices of gay couples and parents.) My focus is on surnaming practices in the United Kingdom and the United States; detailing global and cultural variations in surnaming practices is not possible here (see Regional Organized Crime Information Center 2010).

What are the “always already” existing social contexts (Shilling 2012, p. 4) shaping people’s surnaming practices in the United Kingdom and United States? As I noted earlier, the contemporary legislative frameworks regulating names in these countries are permissive. This non-regulation means that, under common law in both countries, individuals have the right to use any surname as long as there is no fraudulent intent. Yet, in practice, surnaming practices are heavily constrained by cultural traditions (Emens 2007), especially, I argue here, those relating to sex and gender. In the United Kingdom and United States, surname practices are predominantly patronymic (i.e., derived from a male ancestor) and patrilineal. In other words, at marriage to someone whose sex category is male, those whose sex category is female are routinely expected to change from their birth surname to that of their husband. Moreover, children are surnamed after their father.

In the United Kingdom, around 94% of women married to a man change their surname to his. A small minority (4%) use both their surname and their husband’s surname, and 1% “keep” their own surname (Valetas 2001). In the United States, the picture is similar: 90% of women married to a man change their surname to his surname, 5% use a hyphenated surname, 3% use other alternatives, and 2% exclusively use their birth surname (Gooding and Kreider 2010). In the United States, patronymic naming cultures meant that, until as recently as the 1970s, married women could not use their birth surname to vote, to gain a passport or to hold a bank account. Nowadays, either through statutes or case law, all U.S. states allow for married women to retain use of their birth surname (MacDougall 1985). It is clear that, as in the United Kingdom, the vast majority of married women choose not to do so.

Like patronymic surnaming, the patrilineal surnaming of children is an entrenched habit in U.S. and UK societies with similar historical antecedents. Family surname choices favoring the retention of men’s family surnames and the discarding of women’s arose in these countries along with the development of private property rights, modern legal systems, and the expansion of the modern nation-state (Scott et al. 2002). There are no available statistics on surname choices made for children but in a U.S. survey, Johnson and Scheuble (2002) found that less than 3% of children reported on had either their mother’s surname only or a joint surname; the rest had their father’s surname only. In the United States, state laws, either by statute or common law, gave fathers the right to give children their surname. During the 1970s, in the context of the Equal Protection Clause of the Fourteenth Amendment, the automatic right for the father’s surnames to prevail was replaced in many states by seemingly more gender-neutral judicial decisions or statutes (Grossman 2003).

However, in practice, in the United States (as in the United Kingdom) disputes between parents over the surnaming of a child (post-divorce, for example) often continue to be resolved in ways that are not gender-neutral. In particular, the mother’s interest in passing on her surname has not been ranked as highly as the father’s interest in passing on his (Grossman 2003). The perpetuation of family surname choices in which men’s surnames are favored over women’s have been recognized by both the United Nations (1979) and the Council of Europe (2008) as a political issue of (in)equality. Yet, at the time of writing, patrilineal and patronymic surnaming remain ubiquitous cultural traditions in many countries of the world (Regional Organized Crime Information Center 2010).

In addition to being an indicator of inequality in the gender order, what do patronymic and patrilineal surnaming practices typical of the United Kingdom and the United States suggest about bodies, identities, and the doing and redoing of gender? Survey data from the United States suggests that married women who take their husband’s surname are more likely to be White, to have lower levels of educational qualifications, to marry at a younger age, and to endorse more traditional gender-role attitudes compared to women who go against the norm by retaining their birth surname (Goldin and Shim 2004; Gooding and Kreider 2010; Johnson and Scheuble 1995, 1996b). Characteristics associated with non-conventional marital surnaming by women include having higher levels of educational qualifications, a higher age at marriage, more liberal gender-role attitudes, and identifying their race/ethnicity as other than White (Goldin and Shim 2004; Gooding and Kreider 2010; Johnson and Scheuble 1995, 1996b). Evidence suggests, then, an association among surname choices, social class, race/ethnicity, and what might loosely be termed orientations to gender.

Women’s choices about surnames at marriage can be recognized, I argue, as a core part of their embodied doing of gender in adulthood. The great majority comply with patronymic traditions, whereas a small minority, by retaining their birth surname (in whole or in part), creatively re-do gender through resisting the patriarchal norm. Yet, evidence on aspects of the process of choice, such as married women’s rationales for their surname choices, show that it is not just a simple matter of habitual compliance with or creative resistance to patriarchal naming practices. Instead, as I discuss next, surname choices are embedded in multi-stranded and complex strategies relating to embodied sex category and the doing of gender.

A comparative U.S./Russian study by Boxer and Gritsenko (2005) suggests that (heterosexual) women change their surnames at the naming crisis point of marriage for a variety of reasons, such as to signal family connectedness (to show marital union/commitment and family solidarity and/or to mark the beginning of a new stage of life) or as a pragmatic means of avoiding confusion over disparate surnames within a family unit (for the UK, see Thwaites 2013; Wilson 2009). These findings support arguments by scholars that surname choices are linked to family practices of the display of connectedness and affinities, of “We” identities (Almack 2005; Davies 2011; Elias 1991; Finch 2008). Of course, among heterosexual married couples, such outcomes of surname changing could equally be achieved by those in the embodied sex category of men changing *their* surname, but this is not now, nor has it ever been (in the UK and U.S. at least), a practice consistent with the doing of hegemonic masculinity.

At marriage, rather than choosing either surname of the man or (more rarely) the surname of the woman, relatively few heterosexual couples (800 in the UK in 2012) create an alternative by meshing or blending their two surnames together to make an entirely new one (Barnett 2012). There are no available figures for how many men change their surname to that of their wife, but, in contrast to women’s experiences, it remains a rare, non-normative, and less than straightforward process (Emens 2007). A newspaper report by Harris (2008) reveals how difficult it is in reality for a man in the United Kingdom to change his surname to his wife’s at marriage and how people respond with confusion, disbelief, and disapproval if he does.

Instead of married men changing their surnames to that of their wife (in order to display family connectedness and affinities), then, it is instead routine and expected that these outcomes are achieved through women changing their surnames. That embodied sex category and gender are key to surnaming practices is illustrated by my own experience (Pilcher 2016). In 2007, my partner (a man) and I had a civil marriage ceremony (in the UK). At the point of signing the marriage register, the Registrar reminded me not to sign it using my new married name, but in my old surname. Having presumably sex-categorized me on the basis of my bodily appearance as the woman in our heterosexual partnership, the Registrar had made the (erroneous) assumption that I would change my surname as an automatic consequence of marrying.

Evidence from the United States shows that the minority of women who, like me, “keep” their birth surname after marriage do so for reasons either of continuity of professional identity, longevity of their birth surname, feminist ideology or some combination of these (Gooding and Kreider 2010; Johnson and Scheuble 1996a). These findings further support an argument that the surname choices and practices of women “keepers” are important in their creative redoing of gender—they go against the norm and keep their birth surname as part of their identification as someone with equally legitimate claims to surname retention as a man. (Other women may engage in surname practices which, although non-conventional among Whites, reflect their cultural heritage; for example, Latino/a surnames traditionally contain both the father’s and the mother’s paternal family names) (Gooding and Kreider 2010; Regional Organized Crime Information Centre 2010).

In the United Kingdom, the major group of official name changers are women reverting back to their birth surnames after the naming crisis point of divorce (Barkham 2010). This pattern suggests to me that these women are creatively resisting gender norms by (re)claiming their prior gendered embodied named identity. The importance of patronymic and patrilineal naming for doing and redoing gender is also indicated, I argue, by the gender divide between women and men on the issue of surnaming. In essence, evidence suggests that men are more likely than women are to favor the retention of conventional patronymic and patrilineal surnaming practices (in the U.S., Intons-Peterson and Crawford 1985, Scheuble and Johnson 1993a, b, Shafer 2017; in the UK, Wilson 2009). In a UK study, Thwaites (2013) found that some men became very upset if their female partner even considered the idea of not changing her surname at marriage. For me, such findings suggest a link, for some men at least, between adherence to patronymic and patrilineal surnaming practices and the validation of their masculinity.

Studies of the surnaming practices of gay male couples suggest that surname changing is not practiced (Clarke et al. 2008; Patterson and Farr 2016; Suter and Oswald 2003), whereas studies of lesbian couples with children reveal a variance in surnaming practices (Dempsey and Lindsay 2017). In most cases, though, the child is given the biological mother’s surname only, or less often, a joint surname to include that of the social mother (Almack 2005; Gartrell et al. 1999). Overall, though, the limited empirical evidence on gay couples and parents places restrictions on knowledge and understanding of the ways that sexuality may intersect with sex and gender in family and kinship naming practices.

Data from the United Kingdom and the United States on surname practices at marriage and divorce, the surnaming of children, and the characteristics of women making surname choices, along with evidence on a gender divide in support for conventional surnaming practices which privilege men’s surnames over women’s, suggest that patronymic and patrilineal surnaming practices are strongly related to (sex categorized) bodies and the doing of gender. As I have shown, surnames, like forenames, are key to the ways gender differences are routinely accomplished through the everyday, ongoing embodied social interactions that sustain identities. In addition to its doing, surnames can also play a part in the re-doing of gender. Moreover, it is clear that surnaming practices have outcomes which are socially uneven and unequal. As with forenames, the concept of gendered embodied named identities enhances our understanding of these processes via its focus on the relationship among surnames, bodies, and gender.

## Names, Gender Identities, Difference, and Inequalities

From an everyday, common-sense perspective, personal names are merely labels (naming words or proper nouns in grammatical terms) that are applied to individuals and which serve to identify them as such. In my article, I have re-purposed evidence to overcome this functional fixedness about the purpose of names by showing that forenames and surnames are, in addition, doing or action words in relation to sex and to gender. Personal names are key both to the decisive accomplishment of the categorization of sex at birth and, subsequently, in relation to the ongoing management of gender conduct appropriate to sex category. In this final section, I draw together my ideas about the important, specific cultural work that personal names do in relation to embodied sex category and to gender identities, difference, and inequalities, by revisiting three key questions that I identified in my introduction. I summarize the ways our understandings of sexed and gendered naming practices are now enhanced in relation to these questions, including how forenames and surnames are used as multi-faceted tools in the doing of sex and gender.

A key question I set out to address in the present article was how, in relation to sex and gender, embodied individuals respond at critical points of naming in the life course. These critical/crisis points (Shilling 2008) represent, at least in theory, opportunities for individuals to use personal names either in the reproduction of the normative (and patriarchal) gender order, or in its disruption. At the crisis point of giving a forename to a newborn child, evidence presented in my article shows that most people respond normatively. In other words, at birth, a child is typically given a forename which is normatively appropriate for the sex categorization attributed to their body. The choice of androgynous or sex/gender-neutral forenames, which might serve to disrupt the gender order, remain a rarity (Herbert and Aylene 2014; Lieberson et al. 2000).

Evidence discussed earlier showed that gendered forenames are important in the process of doing difference in the context of the power structures of race and ethnicity (Edwards and Caballero 2008; Fryer and Levitt 2003; Sue and Telles 2007). However, such (otherwise creative) naming practices of ethnic difference tend not to disrupt the normative gender order, typically (re)producing binary sex categorizations and, in some instances, the valuing of male/masculinities over female/femininities. For transgender people, choosing a new forename is a critical point in passing in their chosen gender. However, evidence considered here (Connell 2010; Gabbatt 2013; Kessler and McKenna 1978; see also, Schilt 2006) suggests that (non-gender neutral) forename choices made by transgender people can contribute to the reproduction of normative naming practices which habitually link forenames to one or the other of the binary sex categories (female/male) and to the associated display of gender.

Forenames can be conceptualized, then, as routinized, normative tools of categorization, used by individuals in the determination, confirmation, and display of sex category—initially at birth, subsequently throughout the life course, and, for transgender people, at a key point in their redoing of their gender. In being heavily embedded in the categorization of sex and the associated display of gender, forenames can also be recognized as “tools of negation” (to repurpose Connell’s 1987, p. 79–80, concept)*.* In other words, the widespread use of sex and gendered forenames in place of androgynous forenames operates to negate (or downplay) similarities between embodied individuals and accentuate their differences, including of genitalia.

Forenaming practices at key naming crisis points in a person’s life course can also be conceived of as tools of compliance with the doing of sex and gender as binaries. Such practices might be unconsciously (habitually) compliant or be a pragmatic choice, so as to avoid the problems caused by contradictory embodiment, where forenames are perceived as a-typical for a person’s sex category. On the other hand, forenames which are consciously chosen and creatively used by individuals, either because of their gender neutrality or precisely because of their element of contradictory embodiment in relation to sex categorization, can be recognized as tools of resistance to the doing of sex and gender as binaries, as well as a means of disrupting the normative gender order. For those transgender people who identify as gender queer, a creative choice of a forename that is sex- and gender-neutral helps present and authenticate their gender identity as one that does not adhere to the binaries of female/male or feminine/masculine (as shown by the interviewee Agape in Connell’s 2010, study).

Like forenames, surnames operate differently according to embodied sex category and to gender. Marriage and/or the surnaming of children are the key choice crises points at play here, offering individuals an opportunity either to go-with-the-flow of normative surnaming practices in relation to sex and gender or to disrupt them. As with forenames, the evidence shows that most people’s choices are normative, and so the (patriarchal) gender order is (re)produced: patrilineal and patronymic surnaming practices are prevalent in both the United Kingdom and the United States (Gooding and Kreider 2010; Johnson and Scheuble 2002; Valetas 2001). Patronymic and patrilineal family surnaming operate, then, as routinely deployed tools which display that women and children belong to men in a hierarchical gender order. A man’s (embodied) sex categorization invariably means that there are no cultural expectations whatsoever that he should, at marriage to a woman, change his surname to hers; precisely the opposite is true for those whose bodies have been categorized as female. Rejection of such surnaming practices might be interpreted negatively as discrediting an individual’s femininity/masculinity (Harris 2008; Robnett et al. 2016), or, more positively, as a redoing of gender along more equalitarian lines (Mills 2003; Thwaites 2013; Wilson 2009). Surnaming is, then, related to sex categorization, and it is a tool for the display of gender. Like forenaming, surnaming practices are subject to interpretation and evaluation, and they are part of the process whereby gender is determined, attributed, and authenticated in ongoing interaction through practices of embodied named identity.

The majority of women who change their surname at marriage to a man, and/or surname their children after their father, can be recognized as practicing compliance with a gender order which gives greater value to masculinities than to femininities. Such practices might be unconsciously (habitually) compliant, or be a pragmatic choice so as to avoid problems that may arise from having an array of surnames within a family unit. The small minority of women (and, even more rarely, men) who, in heterosexual partnerships and/or marriages, or at the point of divorce, make creative, non-normative surname choices for themselves and/or their children can be conceived of as deploying surnames as tools of resistance to the gender order. As I noted earlier, the reasons (heterosexual) women give for changing/not changing surnames at marriage and/or for the surnaming of their children are multi-stranded and complex (Boxer and Gritsenko 2005), and undoubtedly they are bound up with differential positions of privilege and disadvantage, not least of which relate to social class and education (Goldin and Shim 2004; Gooding and Kreider 2010; Johnson and Scheuble 1995, 1996b). Nonetheless, these practices of gendered embodied named identity are strongly revealing of the centrality of marital and family surnaming as tools related to sex categorization and used in the doing and redoing of gender.

The second of the key questions I identified in the introduction is concerned with how individuals experience and manage crises caused by the breaching of normative expectations about the coincidence of names with sexed/gendered bodies. Evidence on this issue is more limited. In the case of forenames, it would seem that normative expectations about the coincidence of bodies, sex category, gender, and forenames are so strong, that those perceived by others to have inauthentic or wrong forenames may find that their gender conduct is held to account as a consequence (as shown by the experiences of Julie, the male-to-female transperson in Connell’s 2010, study). Cultural knowledge of the strong association between forenames, sex, and gender, then, leads people to use forenames as tools in the attribution and authentication of sex and gender (both for themselves and for others) in responsive and creative ways. Forenames that are gender-wrong can be retained, despite any perceived contradictory embodiment (as in the song, “A Boy Named Sue”; Absolute Lyrics 2017). Nonetheless, such forenames are likely to cause initial and ongoing problems (Figlio 2007) as others deploy strategies to repair their common-sense knowledge about forenames, sex, and gender in the attempt to reconcile the dissonance between the embodied individual they experience and that person’s forename.

The cultural expectations in the United Kingdom and the United States around the links among sex category, marital surnaming, and the surnaming of children mean that assumptions are routinely made by others about women’s and children’s surnames being the same as that of the family’s man. When these normative expectations are breached (when women retain their birth surname at marriage, when men change their surnames at marriage, or in disputes over children’s surnames post-divorce) responses may include confusion and disapproval and/or a fallback to normative understandings (Grossman 2003; Harris 2008; Robnett et al. 2016). Such normative understandings result in the continued deployment of patriarchal and patrilineal surnaming practices in defiance of the preferences of the individual whose surname it is (Davies 2011; Pilcher 2016; Wilson 2009).

The third of my key questions asked how personal naming practices and their outcomes are socially uneven and unequal. Evidence reviewed in my article shows that sex- and gender-categorized forenames remain strongly prevalent over gender-neutral ones for newborns, and they are often important markers for transgender people in helping to secure their chosen gender identification. These are forenaming practices that serve to emphasize normative understandings of the importance of sex and gendered binary differences, rather than commonalities, between embodied humans. Evidence discussed earlier suggests that even when gender-neutral forenames (given to both boys and girls) are in circulation, their deployment for boys gradually decreases (Lieberson et al. 2000); the association (albeit only partial) with lesser-valued femininity is a source of contamination for (originally) androgynous forenames. Likewise, boys with forenames more typically given to girls have been shown to be more disadvantaged than vice versa (Bryner 2010; Figlio 2007), whereas even for girls, female-typed forenames may signal weaker competencies than male-typed forenames in some circumstances (Moss-Racusin et al. 2012). Forenames can be chosen to display ethnic identification and honor ethnic heritage (Edwards and Caballero 2008; Fryer and Levitt 2003), but it seems that such creative choices may at the same time (re)produce sex categorization, gender display, and masculine privilege (Fryer and Levitt 2003; Sue and Telles 2007). In summary, far from being even and equal, forename practices and their consequences are heavily sex- and gender-typed and provide evidence of the continued valuing of masculinities over femininities.

In the case of surnames, identifications based on embodied sex category continue to be the primary determinants of marital surnaming and children’s surnaming, at least among heterosexual couples in the UK and the U.S. (Gooding and Kreider 2010; Johnson and Scheuble 2002; Valetas 2001). The normative choices made by heterosexual women at marriage and about surnames for children have been shown to be multi-stranded and complex (Boxer and Gritsenko 2005; Davies 2011; Nugent 2010), not least in terms of the interplay of privilege and disadvantage in relation to social class, education and ethnicity (Goldin and Shim 2004; Gooding and Kreider 2010; Johnson and Scheuble 1995, 1996b). In summary, well-educated, professional, and other than White women are among those most likely to make the minority choice of retaining their birth surname at marriage. Men rarely change their surname at marriage to a woman. I have argued elsewhere that (White, heterosexual) men have the strongest, most consistent, embodied named identities over their life courses: their (otherwise) privileged social position means they are the least culturally enabled to make (official) changes to their names (Pilcher 2016). Meanwhile, men remain more supportive than women of the continuation of normative surnaming practices at marriage and for children which privilege men’s surnames (Scheuble and Johnson 1993a, b; Shafer 2017; Thwaites 2013; Wilson 2009). In summary, intersectional differences in privilege and power (Hill Collins and Bilge 2016) may impact upon people’s awareness of surnaming options at marriage and for children, and their ability to act upon those options, as well as their support for the continuance of patriarchal and patrilineal practices. Once again, personal naming practices and their outcomes are shown to be socially uneven and unequal.

## Areas for Future Research

The limited attention thus far paid to the topic means that there is plentiful scope for further sociological research to unpick the complexities of forename and surnames choices and uses in relation to sex and gender. The practices of transgender people offer a particularly rich opportunity for exploring the part forenames play in constituting and displaying gender identifications. The family surnaming practices of gay and queer civil-partnered or married couples may offer special insight into how “doing we” through surnames is gendered in complex ways beyond that suggested by evidence on the practices of heterosexual couples. It would be illuminating, too, to gain a greater understanding of the associations that heterosexual men make among surnames, spousal and parental relationships, and masculine identities.

Broader questions to be answered through future empirical research might include how individuals understand the forenames (and surnames) they have been given in terms of the gendered (and racial/ethnic) meanings they encode. Meanwhile, it is still true to say that (in the UK and the U.S. at least) the fact that names are heavily sexed and gendered is likely regarded as a relatively minor feature of contemporary social life—as a trivial concern rather than as a big, pressing social and political issue. Yet, as I have demonstrated in my article, these are not benign or neutral cultural practices. The concept of gendered embodied named identity has great utility in showing how naming practices are, in fact, core to the production and reproduction of binary sex categories and to gendered hierarchies and inequalities, and they are related in important ways to doing difference. Far from being small and insignificant, personal names are powerful cultural tools and deserve greater attention than they have to date, not least of all by gender scholars.
